# *P *elements and MITE relatives in the whole genome sequence of *Anopheles gambiae*

**DOI:** 10.1186/1471-2164-7-214

**Published:** 2006-08-18

**Authors:** Hadi Quesneville, Danielle Nouaud, Dominique Anxolabéhère

**Affiliations:** 1 Dynamique du Génome et Evolution, Institut Jacques Monod, CNRS, Universités P.M. Curie and D. Diderot 2, Place Jussieu, 75252 Paris, France; 2 Bioinformatics and Genomics Lab, Institut Jacques Monod, CNRS, Universités P.M. Curie and D. Diderot 2, Place Jussieu, 75252 Paris, France

## Abstract

**Background:**

Miniature Inverted-repeat Terminal Elements (MITEs), which are particular class-II transposable elements (TEs), play an important role in genome evolution, because they have very high copy numbers and display recurrent bursts of transposition. The 5' and 3' subterminal regions of a given MITE family often show a high sequence similarity with the corresponding regions of an autonomous Class-II TE family. However, the sustained presence over a prolonged evolutionary time of MITEs and TE master copies able to promote their mobility has been rarely reported within the same genome, and this raises fascinating evolutionary questions.

**Results:**

We report here the presence of *P *transposable elements with related MITE families in the *Anopheles gambiae *genome. Using a TE annotation pipeline we have identified and analyzed all the *P *sequences in the sequenced *A. gambiae *PEST strain genome. More than 0.49% of the genome consists of *P *elements and derivates. *P *elements can be divided into 9 different subfamilies, separated by more than 30% of nucleotide divergence. Seven of them present full length copies. Ten MITE families are associated with 6 out of the 9 *P*subfamilies. Comparing their intra-element nucleotide diversities and their structures allows us to propose the putative dynamics of their emergence. In particular, one MITE family which has a hybrid structure, with ends each of which is related to a different *P*-subfamily, suggests a new mechanism for their emergence and their mobility.

**Conclusion:**

This work contributes to a greater understanding of the relationship between full-length class-II TEs and MITEs, in this case *P *elements and their derivatives in the genome of *A. gambiae*. Moreover, it provides the most comprehensive catalogue to date of *P-*like transposons in this genome and provides convincing yet indirect evidence that some of the subfamilies have been recently active.

## Background

Transposable elements (TEs) were undoubtly closely involved in the evolution of the genomic sequences we observe today. TEs can be considered to be parasites, but they all have genome restructuring capabilities and appear to play an important part in genome evolution. The availability of the full genome sequence for a given species now gives us an opportunity to assess the overall impact of TEs on the sequence structures, dynamics and functions of DNA.

In a genome, autonomous TE copies coexist with deleted copies that have frequently been reduced to few hundred base pairs. Because each of them is a potential player in genome dynamics, they all need to be accurately located. Retrotransposons are known to be closely implicated in genome reshaping [1], but Miniature Inverted-repeat Transposable Elements (MITEs) could also be implicated, since they have very high copy numbers and display recurrent bursts of transposition [2,3]. Class II TEs (also known as DNA TEs) have terminal inverted repeats (TIRs) and transpose via DNA intermediates using a "cut-and-paste" mechanism. Autonomous elements encode a transposase that mediates both their own mobility and that of non-autonomous elements that have retained their TIRs. It has been proposed that MITEs could be a particular type of defective class II element [4]. They are known to be small in size, to have short terminal inverted repeats (TIRs), to be present at a high copy numbers, to tend to be inserted near to genes, and to have a high intra-family DNA sequence identity. Moreover, the 5' and 3' subterminal regions of a given MITE family very often show a high degree of sequence similarity with the corresponding regions of a Class II transposable element family [5]. Several such relationships between MITE families and TEs have been reported in monocotyledons and dicotyledons : between the *Hikkoshi *MITE and the *OsHikkoshi *TE [6], the *stowaway *MITE and TC1/mariner TEs [7], and between the *Tourist *MITE and PIF/Harbinger TEs [8]. In *Xenopus *[9] and mosquitoes [10] such a relationship has been proposed to exist between hAT transposons and several MITE families on the basis of similarities betweenTIRs and between target site duplications (TSD). It is now becoming evident that MITEs are non-autonomous DNA elements that originated from DNA transposons which can still mobilize them. It has been shown recently that active transposons can mobilize MITEs. In rice, the Pong and Ping TE families can mobilize mPing MITEs [11]. However, the presence of MITEs and TE master copies able to promote their mobility over a prolonged evolutionary time in the same genome seems to be rare, and has been only reported by Feschotte *et al *[8,12,13]. This is to be expected, since the proliferation of non-autonomous elements would lead to the extinction of the related autonomous elements as a result of a regulation by titration of active amplification, and the selective pressure for MITE immobility [5].

In this paper, we report the presence of *P *transposable elements and related MITE families over a prolonged evolutionary time in the *Anopheles gambiae *genome. The *P *element is an active DNA TE that was first isolated from *D*. *melanogaster *[14] then from *Scaptomyza palida *[15]. Related *P *transposons have now been reported in the genomes of numerous dipteran species [16,17] and in those of several vertebrates; including humans, birds, fish and the prochordean *Ciona intestinalis *[18,19]. Previous work [20,21] has partially described several distant *P *element families in *A. gambiae*. Using a TE annotation pipeline consisting of several programs dedicated to the identification of repeated sequences, we have identified all the *P *sequences in the sequenced *A. gambiae *PEST strain genome. Interestingly, an *in silico *analysis revealed 10 MITE families associated with 6 out of the 9 *P *subfamilies. Surprisingly, one MITE family whose hybrid structure has ends each of which is related to a different *P*-subfamily, suggests a possible new mechanism for their emergence and their mobility. Comparison of their intra-nucleotide diversities and their structures allows us to propose the putative dynamics of their emergence. A Southern blot and PCR analysis showed that these *P *sequences and their related MITEs are present in five distinct laboratory colonies and seem to have been mobile at least in the recent past.

## Results

### Characterization of nine P subfamilies and their master copies

A thorough mining of the PEST genome by the BLASTX program using known *P *transposases identified in Drosophilidae genomes allowed us to identify *P *sequences belonging to the various *P*subfamilies previously reported by Sarkar *et al*. [20] and Oliviera de Carvalho *et al*. [21]. These authors classified the Anopheles *P *elements into 15 clades (P1-P15) using the full length (P1, P2, and P3) and partially homologous *P *sequences. For each clade we have annotated the *P *canonical element, the TIRs and the coding sequence by their similarity to known *P *transposases (Table 1). Note that clades P5, P7, P9 and P10 have been found in other Anopheles species than *gambiae*, so they have not been reported in this study. For the sake of consistency in the *A. gambiae P *sequence identifiers, subfamily names correspond to those of the corresponding homologous sequences (even if only partially homologous) used in the phylogenic tree published by Sarkar *et al*. [20] in which each clade characterizes a subfamily. The name is followed by "-ref" to indicate that this sequence is the canonical one We were not been able to identify any autonomous *P *sequences with terminal inverted repeats and coding capabilities for the P1 and P6 subfamilies. For seven subfamilies, we identified autonomous elements ranging in size from 4178 bp (AgaP13-ref) to 7545 bp (AgaP4-ref) (Table 1). TIRs range from 27 to 31 bp in length. Clades P2, P8, P12, P13 and P15 have quite perfect TIRs (less than 2 mismatches), and clades P3 and P4 display 8 and 6 mismatches respectively. They are all flanked by perfect target site duplications 8 bp in length, as in *D. melanogaster P *elements.

We have shown previously that all the known *P *proteins contain a specific DNA Binding Domain in their NH2 terminal domain, the THAP domain [19]. We used the Framesearch program of the GCG software package [22] to search the anopheline *P *sequences, for this domain, and found a well conserved THAP domain in all putative *Anopheles P *transposases. This finding implies that a correction is called for to the putative protein gene structure of the AgaP2 and AgaP3 elements described by Sarkar *et al*. [20] and reported as P3_AG and P1_AG respectively in the REPBASE UPDATE database [23].

**Figure 1:**
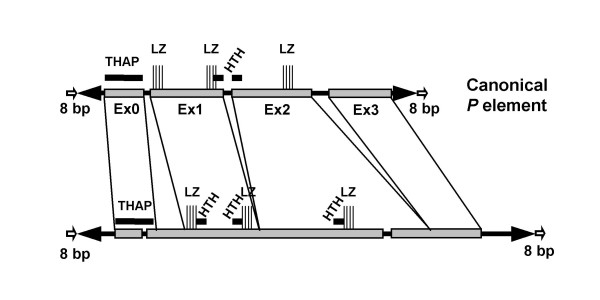
Diagram showing the predicted structure of the *P *element family of *A.gambiae *compared to the structure of the canonical *P *element of *D. melanogaster*. Black triangles represent the TIRs, white arrows represent the TDS, Gray rectangles represent the exons. Protein motifs: THAP: DNA binding domain, LZ: Leucine zipper, HTH: helix-turn-helix

**Figure 2:**
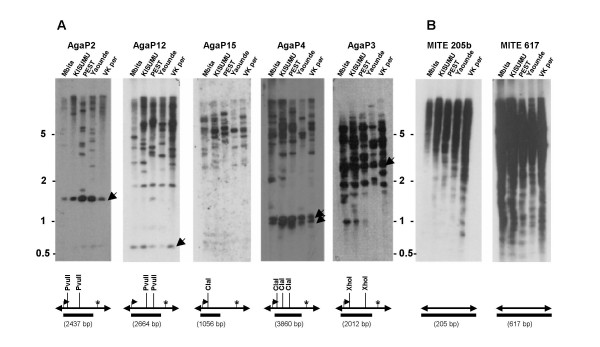
(A) Southern blot analysis showing band polymorphism of five *P *subfamilies in five distinct geographic populations (Mbita, KISUMU, PEST: S molecular type; Yaounde, VK-per: M molecular type). Under each autoradiography, the double arrowed bar corresponds to the reference P sequence, and the thick bar shows the coding region used as a probe (size in parentheses). Exposure 16 hours. The arrows indicate the expected restriction fragments.(B) Detection of AgaP12MITE205b and AgaP15MITE617on the filters previously hybridized by AgaP12 and by AgaP15 respectively, and probed using MITE205 and MITE617 respectively. Exposure 3 hours.

Figure 1 shows the common canonical structure of the *P *element families of *A.gambiae*. The full length elements all have a coding sequence with three putative exons, except for AgaP3-ref, which has an additional putative intron inside exon 2. The following reference elements AgaP2-ref, AgaP3-ref, and AgaP12-ref encode a protein consisting of 879, 895 and 876 amino-acids respectively. The others have stop codons and frame shifts, but all appear to have the ability to encode a transposase when corrected for frame shifts (Fig. 1). All the putative proteins have the characteristic features of *P *transposases: (i) a THAP domain, (ii) a helix-turn-helix motif and (iii) a leucin-zippers motif. If these *P *subfamilies are mobile, we expect that a significant fraction of *P*-containing loci should be polymorphic among geographical distant populations. First, we tested the PEST, KISUMU, Mbita, Yaounde and VK-per laboratory colonies by Southern blots. These laboratory colonies were probed using an internal part of AgaP2-ref, AgaP3-ref, AgaP4-ref, AgaP12-ref and AgaP15-ref copies (Fig. 2a). The restriction digests chosen released a large internal fragment (excepted for AgaP15, see Fig. 2a) that overlapped the coding regions of the copies under test. Each subfamily was present in several copies in all the samples tested; the number of bands is quite similar. In all cases, the expected restriction fragment was detected, suggesting that these colonies probably contain *P *coding elements. The polymorphisms in the banding patterns of the various colonies differed significantly, but their loads in P sequences all seemed to be roughly identical (except for the AgaP2 and AgaP12 subfamilies in the Mbita colony). Consequently, the PEST line can be considered as representative of the *Anopheles gambiae *species with regard to the *P *element subfamilies content in spite of its composite origin (see Discussion section). Since the band polymorphism on its own is not convincing evidence of the current mobility of TEs (or even of recent mobility), we performed PCR experiments to detect insertion site polymorphism for 15 *P *element-containing loci (6 sites for AgaP2, and 3 for AgaP4, AgaP12, and AgaP15) chosen on the PEST sequence. Their presence was checked in DNA samples extracted from the three colonies under test (KISUMU, Yaounde, VK-per) and in the PEST DNA as control. For each insertion, three primers were designed, one specific to the *P *element and two derived from the sequences surrounding the *P *element. An orthologous site gives different PCR amplified fragments when visualized on an agarose gel, depending on whether a *P *copy is present. An example of the results is given [see Additional file 1]. All the loci were empty in the tested colonies, apart from one corresponding to a copy of AgaP15 which also appears to be occupied in the VK-per sample. These findings suggest that these P subfamilies were recently mobile.

### Discovery and characterization of 10 MITE families originated from P element subfamilies

**Figure 3:**
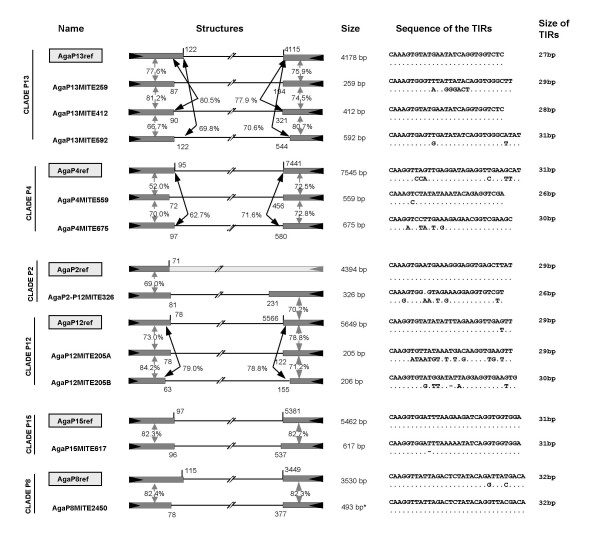
The *P *MITEs and their *P *masters. Diagram of the homologous regions in the reference copy of MITEs and the reference copy of their master. Black triangles are TIRs, gray rectangles are homologous regions between Masters and MITEs. Percentages of identity were calculated using the Bestfit program of the GCG package. Numbers on the edges of the gray rectangles are the coordinates of the region of homology limits. The size is that of the reference copies. Sequence of TIRs: the upper line represents the sequence of the TIR 5' and the lower line, that of TIR 3' (each point is an identical nucleotide, each dash is a gap). GenBank accession numbers : AgaP13MITE259 (DQ301488), AgaP13MITE412 (DQ301489), AgaP13MITE592 (DQ301490), AgaP4MTE559 (DQ301483), AgaP4MITE675 (DQ301484), AgaP2-P12MITE326 (DQ301492), AgaP12MITE205A (DQ301486), AgaP12MITE205B (DQ301487), AgaP15MITE617 (DQ301481), AgaP8MITE2450 (DQ301485). * Due to the heterogeneity of sizes in this family, here is the size of the consensus sequence.

In a preliminary search with our TE annotation pipeline (see Material and methods), we found, among the *P *copies, many hits limited to the same first or last 100 nucleotides, for 6 out of the 7 *P *subfamilies as previously described with TIRs. We searched in the neighborhood of the hits, and found that in many cases there was (i) a sequence similar to the reverse complement of the genomic sequence identified by the hit (a putative TIR), and (ii) a TSD of 8 bp at the boundaries of these putative TIRs. Using this analysis, we identified copies with two TIRs, the 5' and 3' parts of a related *P *element, and an internal sequence not similar to any *P*. This structure suggests that they correspond to a new MITE family that we have called *P*-MITEs. To test this hypothesis, we then searched with BLAST in order to find out whether they are repeated in entirety since if they are, this could be considered to be a genuine *P*-MITE family. For each *P*-MITE family, the set of hits were multialigned using ClustalW to deduce a consensus sequence. Each of the six consensus sequences has TIRs, *P-*element 5' and 3' sequences, and a central region not related to any *P *sequence (Fig. 3). A search in the PEST genome with these MITE consensuses using the TE annotation pipeline identifies numerous full-length copies that are flanked by an 8 bp perfect target site duplication (TSD) characteristic of the *P *element family.

Moreover, the multiple alignments of *P*-MITE copies each related to one of the *P *subfamilies AgaP4, AgaP12 and AgaP13, display two or three subgroups of sequences, each of them corresponding to a distinct MITE subfamily (Fig. 3). Interestingly, we found a composite MITE, AgaP2-P12MITE, that has originated from two *P *subfamilies: the 5'end is related to AgaP2, while the 3' end is related to AgaP12 (Fig. 3).

In summary, we identified 10 *P*-MITE families that we call according to the related autonomous *P *subfamily: AgaP2-P12MITE, AgaP4MITE, AgaP8MITE, AgaP12MITE, AgaP13MITE, AgaP15MITE. Two of them contain 2 subfamilies: (i) AgaP4MITE presents the subfamilies 559 and 675, (ii) AgaP12MITE presents the subfamilies 205A and 205B, and the AgaP13MITE contains 3 subfamilies 259, 412 and 592. All the MITE families share from 114 to 198 bp with their relative *P *autonomous subfamily (the 5' and 3' homologous regions joined), and show identities varying from 52% to 84.2% in these regions (Fig. 3). Conversely, there is no significant identity in the internal regions within either the *P *subfamilies or the *P*-MITE families. This analysis allows us to describe for the first time MITEs derived from *P *transposable elements. The *P*-MITE families copy numbers range from 5 to 310, and their size ranges from 205bp to 2450 bp. Together they constitute up to 0.36% of the genome sequence. In order to evaluate MITE distribution within various colonies, we used two probes corresponding to AgaP12MITE205B and AgaP15MITE617, to re-hybridize the Southern blot filter previously probed with the *P *autonomous subfamilies (Fig. 2b). The strong signals associated with these two probes demonstrate their abundance in the five colonies tested. In what follows, we shall call *P-*master the related *P *autonomous subfamily of a *P*-MITE family, because it is thought to provide the transposase responsible for its mobility.

### Genome wide copy distributions

We searched for the *P *and *P*-MITEs genomic copies by annotating the *A. gambiae *genomic sequences with our TE annotation pipeline (see Method). In order to increase the sensitivity of our detection, we used as the *P *element and *P*-MITE reference sequences, consensus sequences instead of the particular genomic copies described previously as "reference copies". We analyzed the genomic sequence using these consensus sequences (see Methods) as reference sequences, the reference sequences for other known *A. gambiae *TEs and the few unknown repeats previously identified.

Table 2 and 3 summarize the annotations for all the *P-*masters and *P*-MITE families extracted from our genomic survey of the PEST sequence. Table 2 shows for each family: (i) the length of the consensus used, (ii) the number of copies, (iii) the number of copies with lengths differing by no more than 5% from the consensus (as a first approximation, they can be considered here as complete elements), (iv) the number of copies per chromosome arms and the corresponding density in copies per Mb, (v) the median length of the copies, and this length expressed as a percentage of the reference sequence, and (vi) the median percentage of identity with the consensus sequence. Table 3 shows the minimum, maximum and quantiles of the pairwise percentage identity between the genomic copies.

Whatever the *P *subfamily considered, the number of copies per subfamily was small (from 3 to 41) as was the number of complete copies, which ranged from 1 to 6. *P*-MITEs were more numerous (from 44 to 571) than their *P*-master counterparts. They were also less markedly deleted, as shown by their respective median lengths and the proportion of complete copies relative to their total copy number. An obvious explanation is that the longer a sequence, the more random deletion can occur, and so short sequences, such as MITEs, have undergone fewer deletions.

The median pairwise percentage identity (see Table 3) allowed us to estimate how old a family is, as old families are more heterogeneous than recent ones and thus have less pairwise identity. This in turn allowed us to propose a chronology for their amplifications: *P*-MITE amplifications appear to have occurred more recently than the amplifications of the corresponding *P*-masters, as shown by the pairwise percentage identity quantiles. This finding supports the hypothesis that these *P*-MITE families derived initially from their *P*-master. All *P-*master subfamilies show about the same level of heterogeneity (identity from 78.3% to 83.9%) and so can be considered to have the same age, apart from the AgaP8 subfamily that seems to be more recent (90.9%). However, it should be noted that this value is calculated from only 17 copies. Although more recent than their respective *P*-master, the *P*-MITE families display a greater degree of heterogeneity (median pairwise identity from 80.7% to 95.9%) meaning that their transposition activity must have occurred several times over a longer period.

*P*-masters and their MITEs accumulate on the X chromosome (17.6%) compared to the expectation (9.7%) based on the chromosome length and the total copy numbers (goodness of fit *χ*^2 ^= 106.2, 1df, p-value<2.2e-16). However, no significant differences were found between MITEs and *P*-masters regarding their X *versus *autosomal copy number distribution (homogeneity *χ*^2 ^= 0.0087, 1df, p-value = 0.92). However, this distribution differs significantly from that of other TEs (15.4%, *χ*^2 ^= 5.5, 1df, p-value = 0.019). Taken together, these observations suggest that the mobility of both *P *elements and *P*-MITEs are controlled by the same mechanism.

The U chromosome contains unassigned scaffolds (that cannot be located on a chromosome arm), concatenated together in an arbitrary order, like an artificial chromosome. In a whole genome shotgun (WGS), these scaffolds generally correspond to heterochromatic sequences [24,25]. Moreover, the PEST *A. gambiae *strain is outbred, and a number of individuals were sequenced. Therefore, we expected to find polymorphic regions disrupting the assembly. We expected these regions either to be present in several copies in the assembly and/or not to have enough coverage to be assembled at all. Consequently, small contigs would probably correspond to these polymorphic sequences, and we therefore expected to find them on the U chromosome. However, in the light of the very high TE density (a 3 to 6 fold increase) on the U chromosome, we postulate that the vast majority of its sequences are in fact heterochromatic, as it retains a feature specific to heterochromatic sequences: the TE density. Consequently when we compared the U chromosome to other chromosomes, we were in fact comparing heterochromatic sequences with euchromatic ones. The comparison showed that there was more *P*-masters (60%) than *P*-MITEs (40%) in the heterochromatin (homogeneity *χ*^2 ^= 44.5, 1df, p-value = 2.5e-11). If it is assumed that the TEs in heterochromatic regions are the oldest, this observation confirms that *P*-MITEs are more recent than the *P*-masters. Other TEs were significantly less abundant (37%) than the *P*-master (homogeneity *χ*^2 ^= 68.2, 1df, p-value<2.2e-16), and even than *P*-MITEs (homogeneity *χ*^2 ^= 9.23, 1df, p-value = 0.002). This could obviously be explained by a bias toward recent TEs in the discovery of new TE families (they are more abundant and less heterogeneous).

The mean GC% for consensus sequences is 35.1% and 38.1% for *P*-masters and *P*-MITEs respectively. They are AT-rich as this is generally found for TEs [25,26]. For each copy, we determined the GC% of its insertion site, taking 20 bp of the 5' flanking genomic sequence, and 20 bp of 3' flanking genomic sequence. The mean GC% for the *P*-master and *P*-MITEs insertion regions were 40.8% and 43.2% respectively. Compared to the GC% of the genome (44.3%), the insertion preference appears to be biased slightly towards AT-rich regions. The trend is less pronounced for *P*-MITEs than for *P*-masters, but matches what has been reported for other Anopheles MITEs [27].

As expected, very few copies were found in exons, because of their deleterious effect. Table 4 shows TEs occurrences within or around the annotated genes. No differences in the orientation of the TE copy in introns, or in 5' and 3' 500-bp transcript flanking region (compared to the gene orientation) were statistically significant (data not shown). The copy proportion in introns for *P*-MITEs (14.9%) and other TEs (15.4%) were significantly lower (goodness of fit *χ*^2 ^= 8.011, 1df, p-value = 0.005 and *χ*^2 ^= 80.73, 1df, p-value<2.2e-16 respectively) than what would be expected by chance (17%, calculated as the proportion of introns in the genomic sequence). Other MITEs (20.3%) had significantly higher copy proportions (goodness of fit *χ*^2 ^= 45.1, 1df, p-value = 1.9e-11), whereas *P*-master (15.7%) was not significantly different (goodness of fit *χ*^2 ^= 0.31, 1df, p-value = 0.57).

Looking at the 500-bp transcript flanking regions, we can see that *P*-masters and *P*-MITEs were over-represented in the 5' flanking region compared to other TEs (homogeneity *χ*^2 ^= 84.3, 1df, p-value<2.2e-16) or what would be expected by chance (1.1%, calculated as the proportion of the 5' flanking regions in the genomic sequence; goodness of fit *χ*^2 ^= 12.02, 1df, p-value = 5.3e-4). In contrast, we can see that *P*-masters and *P*-MITEs are a clearly under-represented in the 3' flanking region when compared to other TEs (homogeneity *χ*^2 ^= 5.3, 1df, p-value = 0.02) or what would be expected by chance (1.1%, calculated as the proportion of the 3' flanking regions in the genomic sequence; goodness of fit *χ*^2 ^= 22.6, 1df, p-value = 1.9e-6). The 5' preference has already been reported for *P *elements in *D. melanogaster *[28], and seems to be a feature of this TE family. However, the 3' under representation is a new feature. We have compared the 5'-3' distribution of *P*-MITEs with that of other MITES. The 5'-3' distribution of other MITEs is symmetric, and differs significantly from that of *P*-MITEs (homogeneity *χ*^2 ^= 35.13, 1df, p-value = 3e-9).

*P*-MITEs appear to be subject to greater counter-selection in introns and in 3' flanking regions than other TEs. This suggests that their insertions may have a major impact on transcription. Specific features of *P*-MITE sequences might be responsible for this, since *P*-masters behave differently.

## Discussion

### P element diversity

The diversity of *P *subfamilies in the *A. gambiae *genome is intriguing. Firstly, the coexistence of distant *P *subfamilies and the presence of MITEs with their source of transposase in a same genome over a prolonged period is paradoxical. Seven active (or recently active) *P *subfamilies coexist with amino-acid identity percentages ranging from 49.0% to 53.7%. How have these transposases been differentiated in the same genome? They must either have invaded the genome successively after recurrent horizontal transfers, or have evolved from a common ancestral resident sequence, to form several subfamilies producing distinct *P *transposases. Such functional differentiation would have to be driven by specific selective pressures. This assumes that each transposase and/or its associated transposition events provide the element or the host with some specific advantage. Such a selective advantage for the element could be, for instance, that the new variant is able to transpose at higher rate, whereas an advantage for the host could be that the element is rendered less harmful because it transposes less. This antagonism between host and TE fitness is resolved for a TE by a successful invasion of other naïve species that are more permissive toward it. Otherwise, the element would become immobile by transposition repression and then degenerate rapidly as a result of accumulating mutations. Consequently, new variants must arise by mutation and escape the repression mechanism before degenerating. But the speed at which a population reaches a repressed transpositional state, as seen for *D. melanogaster P *element, is rapid (~20 years). There is very little time left for the variant to emerge, and this would give families with near identical ages. The timing compatible with this scenario could not account for the similarities we observed amongst the families. Obviously a new variant could emerge from dead copies, but this appears rather unlikely. Alternatively, a new transposase might benefit the host. It is known that transposable elements harbor a powerful potential repertoire of new functional genetic abilities that are frequently co-opted into host functions. We have recently shown [19] that all *P *transposases have the THAP domain, a DNA binding domain which is also present in many cellular genes, and is widely conserved in animals. In *A. gambiae *the *P *transposases also bear the THAP domain and consequently may also interfere with THAP bearing cellular genes. According to this hypothesis, transposases could have two functions: one function is to mobilize *P *elements, the other, to bind to a specific genomic target which could confer an advantage on the host by altering a host gene function. However, it is unlikely that this kind of evolutionary event could occur recurrently in the same genome sufficiently often to account for such diversity.

The hypothesis of recurrent horizontal transfer events is more likely. There are several pieces of evidence suggesting that horizontal transfer is the main, and perhaps the only source of selective constraint on the *P *element transposase sequence [29,30]. In the *Drosophila *genus, the co-existence of multiple *P *subfamilies in the *saltans *and *willistoni *species groups is probably the result of multiple invasions all over the taxa in several successive horizontal transfer events [30]. The multiple *A. gambiae P *subfamilies could also have resulted from similar recurrent horizontal transfers events.

The second intriguing point is the coexistence of ten *P*-MITE families associated with their mobility factor (the *P*-masters) in a same genome over a prolonged period. This is also remarkable, because of the mutational genetic load associated with the deleterious effect of the mobility of MITEs. A selective constraint would minimize negative effects by reducing the mobility of *P*-MITEs and *P*-masters. One way this could have happened would be a reduction in the transposase-coding copy number. The small number of autonomous *P*elements in the *A. gambiae *genome may reflect this phenomenon. The current status of the *A. gambiae *genome would then correspond to the final invasion phase when *P*-sequences have begun to be tamed.

### The emergence of MITEs

*P*-MITE families share TIRs that are quite similar to these of *P *transposons. This strongly supports the hypothesis that MITEs, or at least their 5' and 3' parts, are derived from class-II TEs, and replicate by "borrowing" the transpositional machinery of autonomous DNA transposons. It should be noted that *P*-MITE and *P*-master copies both have a TSD 8-bp in length, as predicted if the double strand staggered breaks are produced by the same transposase.

Our data are consistent with the hypothesis that *P *elements originate from various *P *subfamilies that have recurrently invaded following horizontal transfers and then spread through *A. gambiae *populations. The pairwise percentage identity values (Table 3), which reflect the invasion burst ages, suggest that each *P*-MITE invasion burst occurred after the invasion of their respective *P*-master. The median pairwise identity between *P*-MITE copies within each family (ranging from 80.7% to 95.9%) shows that *P*-MITE families have differing ages, and have probably emerged successively.

The origin of the MITE internal sequence remains controversial: it may either be derived from the internal part of the master TE followed by nucleotide degeneration, or it may have been copied from an ectopic site by a conversion process consecutive to a double strand break. We have not been able to detect any significant nucleotide similarity between the internal part of *P-*MITEs and the internal sequence of any *P*-master counterpart. Are the divergences between *P*-MITEs and their *P*-masters too ancient to have conserved any detectable similarity? The ranges of their intra subfamily diversity on the one hand, and the *P*-MITEs 5' and 3' sequences similarity with their respective *P-*master on the other hand, suggest that they have emerged recently enough to have been able to retain significant similarity in this central region. This observation rules out the former hypothesis. Terminal regions are known to be essential for transposition, so the internal region may evolve much more quickly than the terminal sequences. But this is only true for a few base pairs. Indeed, the *D. melanogaster *P transposase ^2^binds on ~20 bp [31] close to the TIRs and cleaves in the TIRs. The size of this binding site is small in comparison to the P-master fragments and cannot explain the conservation over ~100 bp.

In contrast, *P *transposon biology looks as if it supports the latter hypothesis. Indeed, *P *elements in *D. melanogaster *are known to transpose *via *a "cut-and-paste" mechanism, leading to a double strand DNA break, which is repaired *via *a gap repair process using the sister chromatid or the homologous chromosome as template [32]. In some cases, it has been shown that ectopic sequences can be used as a template for this repair event [33]. Consequently, *P*-MITEs could have been derived from their *P*-masters by a specific internal deletion retaining the TIRs and their cis-acting DNA sequence required for *P *transposition, but repaired using an ectopic genomic sequence. In addition, if the nucleotide composition of the chimeric element provides a stable, secondary structure, it could acquire a transposition advantage. The AT-richness of *P*-MITEs promotes randomly occurring repeats, and thus contributes to the stability of the secondary structure of *P-*MITEs.

The MITE AgaP2-P12MITE with its two TIRs, which are related to two different *P*-master subfamilies, suggests a third possible mechanism. A MITE could emerge from two *P*-masters located close to each other. This tandem of *P*-copies could transpose en bloc, in what is called a "macrotransposition" [34]. The two initial inserted copies could be partial, but one of them must retain its 5' sequence and the other its 3' sequence, together with the regions they require respectively for transposition. A deletion is likely to occur between these two copies during the transposition process. As a result, a MITE may have appeared, the central region of which corresponds to a part of the genomic region located between the two initial copies. This scenario could explain the formation of AgaP2-P12MITE. Interestingly, this would also suggest that MITE requires two *P*-master transposases, each one bound at one end, in order to be mobilized. This would be the first MITE to have been reported to have such a requirement. Obviously, we cannot rule out the hypothesis that the P-master from which the AgaP2-P12MITE derived had disappeared.

### The PEST genome and the wild genome

One could suspect that the *P*-sequence composition of the sequenced genome of *A. gambiae *is not representative of the species, since the PEST strain is derived from a cross, mixing two molecular forms (M and S forms) associated with a reduction in gene flow between populations. Indeed, the PEST strain was produced by cross-mating an M-form laboratory strain with the field progeny of an S form. Even though the genomic composition of the outbred colony was predominantly derived from the S molecular form, the assembly of the mosquito genome was nevertheless hampered by the presence of multiple haplotypes, and the PEST strain appeared to have a mosaic genomic structure [35]. However, our Southern blot experiments did not detect clear-cut differences when comparing the abundance in *P *sequences between the DNA samples extracted from the PEST strain, and those extracted from the M or S test populations. As a first approximation, we can state that the PEST genome seems to be representative of the *A. gambiae s.s*. wild genome, at least in respect of TE distribution.

### Genome dynamics

*P*-masters account for only 0.13% of the genome, whereas *P-*MITEs account for 0.36%. Despite of their small size, as a result of their sheer numbers, MITEs may contribute more significantly to genome size than their master counterpart. But, how can we explain such a high number of copies, when ectopic recombination is suspected of playing an important role in TE elimination [36,37]? The strength of ectopic recombination as a mechanism of TE copy elimination is obviously linked to the number of copies present. Indeed, as each copy is a potential target for an ectopic exchange, the more copies there are, the more ectopic exchanges can occur. But the size of the copies matters too. Small copies are less likely to be involved in such an exchange [37]. Since MITE sequences are short, they may escape the ectopic exchange reduction process, and thus remain present in large numbers within a genome.

Our analysis of the P- MITEs chromosomal distribution shows the same bias for the X chromosome than *Anopheles P-*elements, an observation that provides further support for a functional relationship between MITEs and the larger coding elements. Bias in chromosomal distribution has been reported both for MITEs and non-*P *DNA transposons in other organisms [38-40]. The X chromosome bias could result from its rotation in the female germline: X chromosomes are found 2/3 of the time in a female lineage versus 1/2 for autosomal chromosomes. Hence, female-specific transposition regulation may change TE densities ratio between X and autosomes. For instance, maternal TE repression inheritance such as that known to occur for *P *element regulation in *D.melanogaster *(called "P-cytotype") can positively select repressor-producing copies to be located on the X [41]. Indeed, X TE copies are more often in a position to produce an effective repressor, because they are transmitted to the offspring *via *the oocyte cytoplasm. A repression mechanism of this type can be more efficient for *P *elements, and thus the bias is more pronounced for *P *elements than for other TEs

### P for transgenesis

Recently, Rasgon and Gould [42] used computer simulation to study the transgene drive mechanism in an *Anopheles *population based on empirical data for *P *transposable element as support. The success of the invasion of the multiple *P *subfamilies and their MITEs throughout the *A. gambiae *genome gives some clues about the ability of *P *transgenic vectors, built from Anopheline *P *elements, to invade mosquito populations. Firstly, it gives some important information about how to construct a transgenic vector from Anopheline *P *elements. The deleted copies of *D.melanogaster P *elements require at least 138 bp at the 5' end and 216 bp at the 3' end for transposition and excision [43]. The 5' and 3' *P *sequences at the ends of *P*-MITEs are in fact less than 100 bp long. This suggests that only 100 bp is required at both ends of Anopheline *P-*transgenes for them to be mobile. This casts a fresh light on the engineering of Anopheline *P *vectors. Studies of the mechanisms implicated in *P *MITE transposition and amplification may help to improve *P *vectors further. Secondly, in *D. melanogaster*, genomic *P *elements are thought to repress the mobility of *P *transgenes that originated from the same *P *element subfamily. The diversity of *P *transposase subfamilies in *A. gambiae *populations would make it possible to find several specific transposase-TIR pair combinations that are helpful for the amplification of such vectors.

## Conclusion

We provide the most exhaustive catalogue to date of *P-*like transposons in the *A. gambiae *genome and present convincing, yet indirect, evidence that some of the subfamilies have been recently active. These elements can be grouped into nine distinct subfamilies of various ages, six of which appear to be related to ten different MITE families. We describe a remarkable genomic state corresponding to the coexistence of MITEs with their mobility factors. *P*-MITEs and *P *elements show the same genomic distribution bias, which provides further support for a functional relationship between MITEs and the larger coding elements. The mutational genetic load associated with the deleterious effect of the mobility of MITEs probably exerts a selective constraint on the transposase-coding copy numbers. The small number of autonomous P elements that we describe in the *A. gambiae *genome is in accordance with this hypothesis and could reflect the final invasion phase when the taming of *P *sequences began.

## Methods

### Genomic DNA extraction

The *A. gambiae *strains KISUMU (Kenya) and VK-per (Burkina Faso) were kindly provided by the LIN Laboratory (UR016, IRD, Montpellier, France). The Yaoundé strain (Cameroon), the Mbita strain (Kenya) and the DNA of the PEST strain were kindly provided by the Biochimie et Biologie Moléculaire des Insectes laboratory, Institut Pasteur, Paris. PEST, KISUMU and Mbita are classified as the S molecular form, and VK-per and Yaoundé as the M molecular form, according to the classification system based on X-linked rDNA repeat units that recognizes two molecular forms, known as M and S [44,45]. This classification has to be taken in account, because the two forms reduce the gene flow between the populations of the species [46]. The DNA was extracted from frozen adults as previously described for Drosophila [47].

### DNA blot analysis

DNA blot hybridizations were performed on 10 μg of DNA per lane according to standard protocols (Maniatis, Fritsch and Sambrook 1982). Each blot contained DNA from the five laboratory colonies described above. The DNAs were digested either once with PvuII, or double digested with ClaI and XhoI. These digests released large internal fragments overlapping the coding regions of the *P *subfamilies under test. In order to make it possible to compare the patterns, each gel was bi-transferred as recommended by the supplier onto a nitrocellulose membrane (Schleicher and Schuell). The same samples were thus successively hybridized with different *P *subfamilies after dehybridization. The probes used were synthesized by Polymerase Chain Reaction (PCR) from PEST strain DNA templates, and labeled with ^32^P-dCTP using the High-Prime random priming kit (Amersham). The primer sequences and PCR program are available on request.

### *In silico *analysis

#### Genomic sequences

The tenfold whole genome shotgun assembly "MOZ2" of the *A.gambiae *PEST strain was downloaded from the Ensembl web site [48]

The gene annotations were downloaded from the UCSC Genome Bioinformatics web site [49]

#### TE databases

The TE reference set used was derived from the *A. gambiae *RepeatMasker repeat library [50] (A.F.A. Smit, R. Hubley & P. Green RepeatMasker March 6, 2004), multiple alignments from Tu [27], and sequences from Eiglmeier *et al *[51]. In the RepeatMasker repeat library LTR retrotransposable elements are divided into LTR sequences (present on both side of the elements) and internal parts (the regions between the two LTRs). We reconstructed full length LTR retrotransposable elements from this library by adding the LTR sequence on both sides of the internal part. We derived consensus sequences for the MITEs described in Tu [27] from the multiple alignments deposited in EMBL alignment database (accession nos. DS43373-DS43385). A consensus sequence for the Indy element (a non-autonomous LTR retrotransposable element) has been derived from a multiple alignment of the genomic copies found in Eiglmeier *et al *[51].

#### Annotation pipeline

Transposable elements were annotated using the RMBLR procedure from the TE annotation pipeline described by Quesneville *et al*. [52]. Then, consecutive fragments on both the genome and the same reference TE were automatically joined if they were separated by a sequence of which more than 80% consisted of other TE insertions (in this case, we have a nested TE). Note that the TE reference set used to find the *P *element copies also includes other known TEs of *A. gambiae*, which makes it possible to join correctly distant fragments due to nested TE insertions. Otherwise they were taken to be joined if they were separated by a gap of less than 5000 bp or by a mismatch region of 500 nucleotides.

Simple repeats were found using the Tandem Repeat Finder program [53] and used to filter out spurious hits. All TE annotations that were less than 20 bp after removing any regions that overlapped simple repeat regions were eliminated.

#### Consensus building

To build consensus sequences we first searched *P *genomic copies using the *P *"reference copy" sequences (described earlier, see Table 1). For each subfamily, we constructed multiple alignments of the *P *copies (see below). Alignments were visually inspected. For some families, many hits were restricted to a particular region of the reference sequence, beginning and finishing at the same nucleotide position. This happened when the reference sequence contained an unidentified repeat that was also found at many other positions within the genome, but without the flanking sequences of the reference sequence. When this occurred in the internal part of the element, we manually edited the alignment to remove the columns corresponding to the repeat. In a few cases, a TIR region was included in a tandemly or dispersed unknown repeat. In these cases, we chose to add the unknown repeat (containing the TIR) to the reference set used to annotate the genome, so that MATCHER would assign these hits as not belonging to a *P *subfamily. For a few subfamilies, some hits were restricted to a short alignment region with fuzzy limits. When these regions corresponded to a much-degenerated microsatellite (too degenerate to be detected by TRF), we removed the columns that corresponded to these spurious hits. For each multiple alignment, we derived a consensus by selecting the majority nucleotide at each column position. Note that consequently the size of the consensus sequences differs slightly from those of the "genomic reference copies". This is particularly true for AgaP8MITE2450 whose copies are heterogeneous in size due to several independent insertions.

#### Statistical analysis

Statistical analyses were performed using the R software environment [54].

#### Multiple alignments

Multiple alignments for each family were obtained by first computing the pairwise global alignment with the reference sequence using the *gap *program [55], and second by stacking all pairwise alignments to obtain a multiple alignment. Any sequences not present in the reference sequence were removed from the multiple alignments. The consensus was then obtained from a multiple alignment using a simple majority rule.

## Authors' contributions

HQ, DN and DA participated in the conception, design, analysis, interpretation of data, and drafting of the manuscript. All authors read and approved the final manuscript.

## Supplementary Material

Additional File 1 Illustration of the insertion site polymorphism of P transposable elements in Anopheles gambiae populations. Five insertion site of AgaP2 subfamily copies localized in the genomic sequence of PEST. Agarose gel showing PCR products visualized by ethidium bromide and obtained from DNA of four distinct populations: PEST, KISUMU, VKper and Yaoundé. Click here for file

## Figures and Tables

**Table 1:** Master *P *elements in the PEST *Anopheles gambiae *genome

Clades	P master name	TIR (bp)	Nucleotide Length (nt)	Proteine Length (AA)		Genbank Accession Numbers
P2	AgaP2-ref	29	4394	879		DQ301496
P3	AgaP3-ref	31	4949	894		DQ301492
P4	AgaP4-ref	31	7545	883		DQ301493
P8	AgaP8-ref	32	3530	Not coding		DQ301497
P12	AgaP12-ref	29	5649	877		DQ301494
P13	AgaP13-ref	27	4178	885*		DQ301495
P15	AgaP15-ref	31	5462	873		DQ301491

*Consensus sequence

**Table 2:** *P *element and *P*-MITE families copies distributions

**family**	**len.**	**Copy number**		**chrX (22.1 Mb)**		**chr2L (48.8 Mb)**		**chr2R (62.7 Mb)**		**chr3L (41.3 Mb)**		**chr3R (53.3 Mb)**		**chrU (59.6 Mb)**		**med len.**	**med. len. %**	**med. cons. id.**
		**all**	**Compl.**															
AgaP12cons	5329	41	1	3	*0.14*	2	*0.04*	5	*0.08*	8	*0.19*	6	*0.11*	17	*0.29*	76	1.4	88.2
AgaP12MITE205Acons	205	496	310	70	*3.16*	61	*1.25*	36	*0.57*	51	*1.24*	47	*0.88*	231	*3.88*	205	100.0	98.0
AgaP12MITE205Bcons	206	413	286	53	*2.39*	56	*1.15*	64	*1.02*	36	*0.87*	32	*0.60*	172	*2.89*	206	100.0	98.4
AgaP13cons	4105	35	1	1	*0.05*	2	*0.04*	5	*0.08*	1	*0.02*	1	*0.02*	25	*0.42*	290	7.1	89.5
AgaP13MITE259cons	259	173	95	23	*1.04*	21	*0.43*	24	*0.38*	12	*0.29*	12	*0.23*	81	*1.36*	258	99.6	97.7
AgaP13MITE412cons	412	193	66	8	*0.36*	36	*0.74*	48	*0.77*	32	*0.78*	36	*0.68*	33	*0.55*	311	75.5	88.9
AgaP13MITE592cons	592	143	33	1	*0.05*	21	*0.43*	34	*0.54*	28	*0.68*	29	*0.54*	30	*0.50*	339	57.3	89.2
AgaP15cons	4876	34	2	2	*0.09*	0	*0.00*	3	*0.05*	4	*0.10*	6	*0.11*	19	*0.32*	122.5	2.5	90.3
AgaP15MITE617cons	617	571	238	79	*3.57*	59	*1.21*	49	*0.78*	48	*1.16*	48	*0.90*	288	*4.83*	541	87.7	95.3
AgaP1cons	3064	41	4	1	*0.05*	3	*0.06*	1	*0.02*	2	*0.05*	0	*0.00*	34	*0.57*	177	5.8	86.4
AgaP2cons	4159	34	6	1	*0.05*	5	*0.10*	3	*0.05*	4	*0.10*	5	*0.09*	16	*0.27*	458.5	11.0	91.8
AgaP2P12MITE326cons	326	132	58	10	*0.45*	21	*0.43*	22	*0.35*	7	*0.17*	9	*0.17*	63	*1.06*	275	84.4	93.9
AgaP3cons	4822	41	2	2	*0.09*	3	*0.06*	3	*0.05*	5	*0.12*	3	*0.06*	25	*0.42*	223	4.6	88.6
AgaP4cons	6314	58	4	9	*0.41*	4	*0.08*	3	*0.05*	6	*0.15*	1	*0.02*	35	*0.59*	361.5	5.7	81.3
AgaP4MITE559cons	559	218	80	14	*0.63*	33	*0.68*	46	*0.73*	28	*0.68*	35	*0.66*	62	*1.04*	455.5	81.5	92.0
AgaP4MITE675cons	675	187	49	5	*0.23*	29	*0.59*	43	*0.69*	23	*0.56*	39	*0.73*	48	*0.81*	442	65.5	91.5
AgaP6cons	905	3	0	0	*0.00*	1	*0.02*	0	*0.00*	0	*0.00*	0	*0.00*	2	*0.03*	133	14.7	95.5
AgaP8cons	3510	17	1	3	*0.14*	2	*0.04*	0	*0.00*	1	*0.02*	0	*0.00*	11	*0.18*	511	14.6	93.6
AgaP8MITE2450cons	493	44	5	6	*0.27*	5	*0.10*	1	*0.02*	1	*0.02*	2	*0.04*	29	*0.49*	224	45.4	95.4
*P*-masters		304	21	22	*0.99*	22	*0.45*	23	*0.37*	31	*0.75*	22	*0.41*	184	*3.09*			
*P*-MITEs		2570	1220	269	*12.15*	342	*7.01*	367	*5.85*	266	*6.44*	289	*5.42*	1037	*17.41*			
All *P*		2874		291	*13.14*	364	*7.46*	390	*6.22*	297	*7.19*	311	*5.84*	1221	*20.50*			
No *P*		43938		4245	*191.69*	5998	*122.92*	5919	*94.36*	5227	*126.61*	6141	*115.28*	16408	*275.45*			

**Table 3:** *P *element and *P*-MITE intra-families pairwise identity percentages distribution

Family	min	Q5	Q25	median	mean	Q75	Q95	max
AgaP12cons	33.3	66.7	73.6	80.0	80.2	86.8	97.6	100
AgaP12MITE205Acons	33.3	80.8	91.7	95.9	93.6	97.3	98.8	100
AgaP12MITE205Bcons	33.3	79.0	91.0	95.6	93.2	97.6	99.5	100
AgaP13cons	56.3	65.1	75.0	81.8	82.5	92.8	98.2	100
AgaP13MITE259cons	40.0	75.9	85.4	93.8	90.8	96.9	99.2	100
AgaP13MITE412cons	25.0	71.2	76.7	80.6	81.8	86.6	95.1	100
AgaP13MITE592cons	33.3	69.9	78.0	83.3	82.7	88.0	94.1	100
AgaP15cons	60.7	71.1	79.2	83.9	85.1	91.9	99.9	100
AgaP15MITE617cons	33.3	74.5	82.7	89.1	87.4	92.8	96.3	100
AgaP1cons	46.2	60.0	74.4	80.0	81.6	90.2	100	100
AgaP2cons	45.0	65.1	72.3	81.6	83.0	97.3	100	100
AgaP2P12MITE326cons	44.4	76.2	84.1	88.9	87.8	92.6	96.0	100
AgaP3cons	46.7	61.5	74.7	82.5	83.5	96.0	100	100
AgaP4cons	38.5	61.0	72.7	78.6	79.6	89.6	99.2	100
AgaP4MITE559cons	33.3	73.6	79.9	85.0	84.8	89.8	96.6	100
AgaP4MITE675cons	33.3	71.5	78.6	83.0	82.8	88.0	92.4	100
AgaP6cons	95.5	95.5	95.5	95.5	95.5	95.5	95.5	95.49
AgaP8cons	50.0	67.9	87.5	90.6	88.2	92.5	97.2	98.83
AgaP8MITE2450cons	66.7	80.7	89.5	92.6	91.6	95.5	100	100

**Table 4:** TE and MITE locations.

	*P*-master	*P*-MITEs	Other TEs	Other MITEs
Exon	0 (0%)	1 (0.04%)	117 (0.2%)	2 (0.03%)
Intron	48 (15.7%)	383 (14.9%)	6762 (15.4%)	1174 (20.3%)
Same orientation	26	178	3351	589
Opposite orientation	22	205	3411	585
5' flanking	2 (0.6%)	49 (1.9%)	165 (0.3%)	37 (0.64%)
Same orientation	1	27	77	18
Opposite orientation	1	22	88	19
3' flanking	0 (0%)	5 (0.19%)	190 (0.4%)	29 (0.5%)
Same orientation	0	3	88	10
Opposite orientation	0	2	102	19
Other regions	254 (83.6%)	2132 (83%)	36704 (83.5%)	4536 (78%)
Total number	304	2570	43938	5778
