# Supplementary material for: P elements and MITE relatives in the whole genome sequence of Anopheles gambiae
Source: BMC Genomics. 2006 Aug 18;7:214. doi: 10.1186/1471-2164-7-214 (PMC1562414; doi:10.1186/1471-2164-7-214)
Supplement: Additional File 1 — Illustration of the insertion site polymorphism of P transposable elements in Anopheles gambiae populations. Five insertion site of AgaP2 subfamily copies localized in the genomic sequence of PEST. Agarose gel showing PCR products visualized by ethidium bromide and obtained from DNA of four distinct populations: PEST, KISUMU, VKper and Yaoundé. [file 1471-2164-7-214-S1.pdf]

Additional File 1

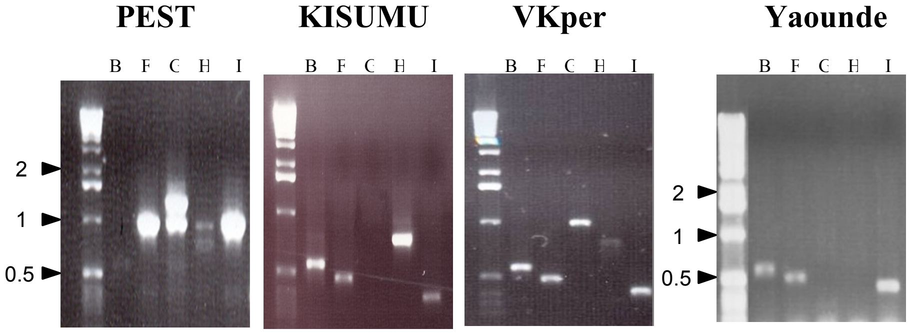

Illustration of the insertion site polymorphism of *P* transposable elements in *Anopheles gambiae* populations

Five insertion site of AgaP2 subfamily copies localized in the genomic sequence of PEST. Agarose gel showing PCR products visualized by ethidium bromide and obtained from DNA of four distinct populations: PEST, KISUMU, VKper and Yaoundé.

Copy	Insertion site coordinate*	Primers	Anneal. T°	PCR Anneal. T°		Amplified Length	
				PEST	others	Occupied site	Empty site
All		Nested primer : GGGGCTGTCATATTCGATGGCTCG	64°C				
B	Chr 2R: 35522531	fl 5' - GGCCAACCGGCGACTAAAACC<br>fl 3' – TCACCCTTACCTTTGCCGGTGG	64°C<br>65°C	64°C	59°C	837 bp	555 bp
F	Chr 2L: 9639890	fl 5'- GGTGTTGTTCCCCTCCGTTTCC<br>fl 3' – TGCCGCAAAACGTCAAAGCTTC	60°C<br>61°C	60°C	55°C	975 bp	476 bp
G	Chr 3R: 51957269	fl 5' - GTCCTGCTTTGGCGTATCGGGG<br>fl 3' – CACACTGGGTCAAAAATCGCGC	63°C<br>61°C	61°C	56°C	1307 bp	991 bp
H	Chr 3R: 48892255	fl 5' - AGCGCATGCTTCCGTCAGCCCC<br>fl 3' – GGGGGCGAGGAACAGAGAGGCG	68°C<br>67°C	64°C	59°C	955 bp	777 bp
I	Chr 3L: 30318829	fl 5' - ATCGCGTGCGGTTTTGTCG<br>fl 3'– TGTCGTCCGGTTTGGGGCGTTG	62.6°C<br>67.2°C	64°C	59°C	1000 bp	399 bp

fl 5': primer designed in the 5' genomic flanking sequence; fl 3' : primer designed in the 3' genomic flanking sequence
* Assembly MOZ 2a

PCR conditions:

For each copy (B, F, G, H, I), 3 primers were designed: two chosen from the genomic sequence flanking the copy and one nested. A PCR reaction contains the 3 primers.

Annealing temperature, specific to each set of primers, is applied for PCR on PEST and Kiss DNA (S molecular type), but decreased of 5°C when applied to Vk-per and Yaoundé DNA (M molecular type). This modification turned out to be necessary because of the nucleotide divergences between these molecular type (della Torre et al. 2001. Insect Mol. Biol. 10 (1), 9-18.; Gentille, G. et al. 2004. Insect Mol. Biol. 13(4) 371-377).

Temperature cycling parameters: First 94°C 3 min, then 30 cycles as follows: denaturation: 94°C 1 min, annealing: depending on the set of primers (see Table below) 1 min, elongation: 72°C 1min30 and final cycle of 72°C for 10 min.
